# Analysis of reciprocating system real-time torque variation during root canal preparation

**DOI:** 10.1186/s13104-019-4080-z

**Published:** 2019-01-21

**Authors:** Flares Baratto-Filho, Jéssica Vavassori de Freitas, Marilisa Carneiro Leão Gabardo, Flávia Sens Fagundes Tomazinho, Jardel Francisco Mazzi-Chaves, Manoel Damião de Sousa-Neto

**Affiliations:** 1 School of Health Sciences, Universidade Positivo, Rua Prof. Pedro Viriato Parigot de Souza 5300, Curitiba, Paraná 81280-330 Brazil; 2 Department of Restorative Dentistry, Universidade de São Paulo, Avenida do Café s/n, Ribeirão Preto, São Paulo 14040-904 Brazil

**Keywords:** Molars, Root canal preparation, Torque

## Abstract

**Objective:**

This study monitored real-time torque variation of the WaveOne Gold (WOG) and Reciproc Blue (RB) during root canal preparation of mandibular molars. Thirty-six mandibular molars were prepared with WOG Primary 25.07 (WOGP, n = 36) and the RB R25 25.08 (RBR25, n = 36) for the mesial canals, whereas WOG Large 45.05 (WOGL, n = 18) and RB R40 40.06 (RBR40, n = 18) for the distal. Canal preparation was divided into thirds and the torque, maximum torque and time, were recorded.

**Results:**

The RBR25 instruments exhibited higher maximum torque in the apical third in contrast to the WOGP instruments (p < 0.05). The intragroup analysis found a significant difference in maximum torque between the cervical and apical thirds, and the middle and apical thirds (p < 0.05) for both instruments (RBR25 and WOGP). The WOGP group had the shortest preparation time (p < 0.05). There were no significant differences between the WOGL and RBR40 for any of the parameters evaluated (p > 0.05). The RBR25 had the highest torque when compared to the WOGP. Both instruments exhibited higher torque in the apical third and there were no significant differences between the instruments in the distal canal.

## Introduction

In recent years the use of instrumentation systems has gained prominence in endodontics, as they require only a single instrument for root canal mechanical preparation. The WaveOne Gold (WOG) (Dentsply Sirona, Ballaigues, Switzerland) and Reciproc Blue (RB) (VDW, Munich, Germany) files are manufactured with M-Wire alloys and have specific heat treatments that provide greater flexibility and fatigue resistance [1–3].

However, instrument fracture during root canal preparation remains a constant concern for endodontists. rotational speed, torque, alloy type, instrument design, instrumentation technique, and operator experience may be directly related to endodontic instrument fractures [4–8].

Electric endodontic motors with torque control, which only release the amount of mechanical energy required for instrument operation, can control these factors [9]. Among the electric models, the X-Smart IQ (Dentsply Sirona, Ballaigues, Switzerland) enables real-time torque monitoring (RTTM), which displays the torque as the instrument is used to prepare the canal; thus, it increases the confidence and safety of professionals during biomechanical preparation, especially when using single-use instruments.

Although these instruments have been widely studied, torque variation during root canal preparations using WOG and RB systems has not been investigated, to our knowledge. Thus, the aim of this study was to compare, ex vivo, the torque of these two reciprocating systems during mandibular molar canal preparations.

## Main text

### Methods

#### Selection of teeth

Thirty-six recently-extracted mandibular molars were used, following approval by the Universidade Positivo Institutional Research Ethics Committee (opinion 2,077,489). All donors signed an informed consent form and a donation term. The teeth were radiographed and analyzed under operative microscopy. Radiographically all teeth had similar canal diameter and morphology. The inclusion criteria were: complete rhizogenesis; presence of three independent canals; and the absence of endodontic treatment and caries, resorptions, perforations, or signs of fractures and cracks. Teeth with radiographic evidence of calcifications and aberrant canal morphology were excluded.

#### Biomechanical preparation of root canals

The biomechanical preparation of root canals was performed by a single operator, an Endodontist with 5 years of experience, aiming to minimize the operator bias.

After coronal access, a #10K-file (Dentsply Sirona, Ballaigues, Switzerland) was used to confirm patency. Once the actual tooth length was determined by visualizing the instrument in the foramen, the working length (WL) was considered 1 mm smaller than this measurement. The canal preparations were performed by thirds to record the torque used in each region.

The mesial canals were prepared using WaveOne Gold Primary (WOGP, Dentsply Sirona, Ballaigues, Switzerland) and Reciproc Blue R25 (RBR25, VDW, Munich, Germany). Thus, the mesial roots were prepared using both systems, alternating their distribution in the mesiobuccal and mesiolingual canals, resulting in 36 samples per group. When necessary, the mesial reference edge was adjusted to ensure the same WL was used for both mesial canals.

The distal canals were prepared using WaveOne Gold Large (WOGL) and Reciproc Blue R40 (RBR40). For this root, the distribution of the systems was alternated between teeth, resulting in 18 samples per group.

The instruments were powered by an X-Smart IQ motor (Dentsply Sirona, Ballaigues, Switzerland), enabling RTTM. The WaveOne instruments were used in the “WaveOne” programming mode and the Reciproc Blue instruments in the “Reciproc Blue” mode. Before the use of each instrument, each system was calibrated using the X-Smart IQ program. One operator performed the instrumentation, and the instruments were discarded after one use.

Irrigation was performed 3 mm below the WL during instrument kinematics with 2.5% sodium hypochlorite using a NaviTip #20 needle (Ultradent, South Jordan, Utah, USA) connected to a 5 mL disposable syringe, followed by a final irrigation with 5 mL of 17% EDTA. The canals were then irrigated with 5 mL of distilled water and dried with absorbent paper tips.

The X-Smart IQ motor enables RTTM. After treatment completion, the motor generates a final report displaying the torque of each movement during modeling. The torque values for each movement, the maximum torque value for each instrument, and the time (s) for each canal preparation were recorded. To eliminate interference or induction of the results, the operator did not have access to the iPad Mini screen (Apple Inc., Cupertino, California, USA) that displayed the real-time torque values.

#### Statistical analysis

The Kolmogorov–Smirnov test was performed to verify the normality of the sample. An independent analysis of the mesial and distal canals by non-parametric testing was performed using the SPSS^®^ (IBM^®^ SPSS^®^ Inc., Chicago, USA), version 24, with a 5% significance level. The Kruskal–Wallis test was used for intragroup analysis to determine significant differences in torque and maximum torque reached in each third of the canal with the same instrument. The Mann–Whitney U test was used for intergroup analysis to determine significant differences in torque and maximum torque among the instruments used within the same third of the canal. The Mann–Whitney U test was also used to determine significant differences in root canal preparation times between instruments.

### Results

The sample was not normally distributed (p < 0.05). The results of the comparisons are described below.

#### Mesial canal (WOGP vs. RBR25)

The mesial canal results are shown in Table 1.

**Table 1:** Median values for torque and maximum torque reached during mesial root canal preparation using WOGP and RBR25 files

Instrument	Third	Median of torque Ncm (min–max)	Median of maximum torque Ncm (min–max)	Time (s)
WOGP	Cervical	0.22 (0.20–0.25)	0.30 (0.30–0.30)^A,a^	53.5 (21–106)
	Middle	0.20 (0.10–0.80)	0.55 (0.10–1.40)^A,b^	
	Apical	0.30 (0.10–0.65)	0.95 (0.20–1.90)^B,c^	
RBR25	Cervical	0.15 (0.10–0.60)	0.50 (0.30–0.60)^A,a^	61.5 (20–107)
	Middle	0.20 (0.10–0.80)	0.60 (0.10–1.30)^A,b^	
	Apical	0.30 (0.10–0.60)	1.25 (0.40–2.20)^B,d^	

Different letters indicate statistically significant differences (p < 0.05). Capital letters indicate comparisons between thirds using the same instrument (intragroup analysis). Lowercase letters indicate paired comparisons between instruments in the same third (intergroup analysis)

#### Intragroup torque analysis by thirds (cervical, middle, apical)

The intragroup analysis by thirds found no significant differences (p > 0.05). Maximum torque was significantly different between regions for both instruments (p < 0.05).

#### Intergroup torque analysis by thirds (WOGP vs. RBR25)

There were no significant differences in torque between groups (p > 0.05). However, maximum torque was significantly different in the apical third between groups (p < 0.05).

#### Preparation time analysis (WOGP vs. RBR25)

Preparation time for the WOGP was significantly shorter when compared to that of the RBR25 (p < 0.05).

In the torque analysis, there were no significant intragroup differences for either the WOGP or RBR25 (p > 0.05). There were also no significant intergroup differences for the cervical, middle, and apical thirds (p > 0.05).

In the maximum torque analysis, there were significant intragroup differences for both the WOGP and RBR25 (p < 0.05) groups. There were also significant intergroup differences for the apical third (p < 0.05).

**Fig. 1:**
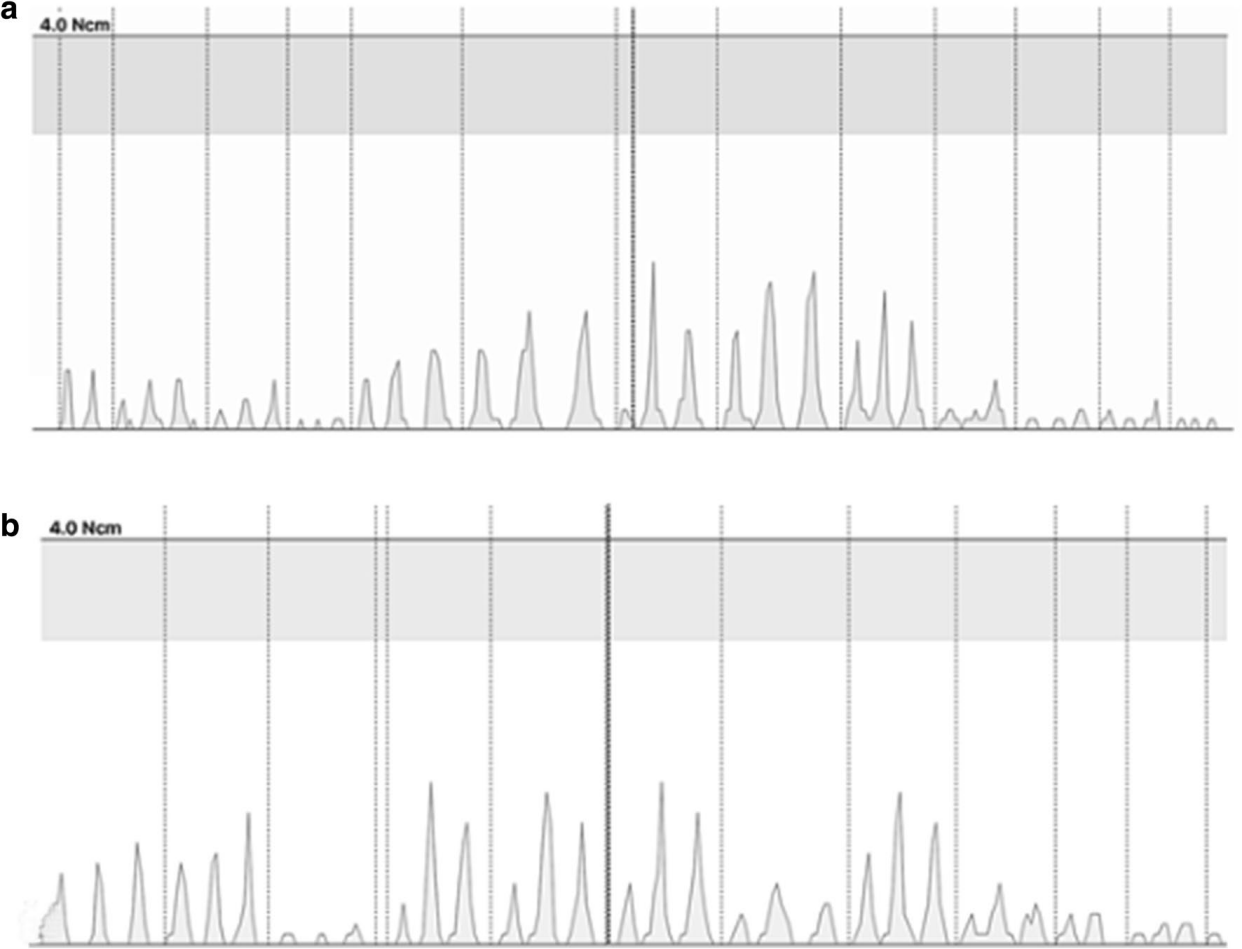
Torque peaks recorded during mesial root canal preparation. RTTM during preparation with WOGP (**a**) and RBR25 (**b**)

In the time analysis, there was a significant difference between instruments (p < 0.05). Figure 1 shows the RTTM during the mesial canal preparations with WOGP and RBR25.

#### Distal canal (WOGL vs. RBR40)

The distal canal results are shown in Table 2.

**Table 2:** Median values for torque and maximum torque reached during distal root canal preparation using WOGL and RBR40 files

Instrument	Third	Median of torque Ncm (min–max)	Median of maximum torque Ncm (min–max)	Time (s)
WOGL	Cervical	–	–	30.5 (13–62)
	Middle	0.15 (0.10–0.50)	0.30 (0.10–0.70)	
	Apical	0.12 (0.10–0.70)	0.55 (0.10–1.70)	
RBR40	Cervical	0.15 (0.10–0.20)	0.20 (0.10–1.0)	27.5 (8–82)
	Middle	0.15 (0.10–0.50)	0.17 (0.10–0.30)	
	Apical	0.17 (0.10–0.30)	0.65 (0.10–2.10)	

#### Intragroup torque analysis by thirds (cervical, middle, apical)

There were no significant intragroup differences in torque (p > 0.05) or maximum torque (p > 0.05).

#### Intergroup torque analysis by thirds (WOGL vs. RBR40)

There were no significant differences between instruments for the middle (p > 0.05) and apical thirds (p > 0.05). The WOL did not generate torque during preparation of the cervical portions; therefore, comparisons for the cervical third could not be made. The RBR40 touched the cervical third in only four samples.

#### Preparation time analysis (WOGL vs. RBR40)

There were no significant differences in preparation time between instruments (p > 0.05).

No torque or maximum torque was recorded for the cervical third while using the WOGL.

In the torque analysis, there were no significant intragroup differences for either the WOGP (p > 0.05) or RBR40 (p > 0.05) group. There were also no significant intergroup differences for the middle or apical thirds (p > 0.05).

In the maximum torque analysis, there were no significant intragroup differences for either the WOGL or RBR40 (p > 0.05) group. There were also no significant intergroup differences for the middle or apical thirds (p > 0.05).

In the time analysis, there was no significant difference between instruments (p > 0.05).

### Discussion

With the development of the X-Smart IQ endodontic motor, torque monitoring for rotating and reciprocating systems has been improved to real time. This allows the clinician to assess variations that occur during root canal preparations and modify the instruments actions.

In vitro methods are typically used for mechanical property analysis of endodontic instruments [3, 10, 11]. However, this ex vivo study better approximates clinical procedures, as it utilizes recently-extracted human. This study compared the torque of two reciprocating M-Wire files, WOG and RB, which were chosen based on similarities in manufacturing, tip size, taper, and kinematics. Although the root canal anatomy may make sample homogenization difficult, the bias of this variable was reduced by using the WOG and RB instruments in the same root and alternating the canals; therefore, both instruments performed similar actions on the same root length and curvature.

The median torque used during canal preparation was not significantly different between the instruments tested, meaning that both instrument systems exhibited similar behavior. However, there was a significant difference between instruments for the torque used during the apical third preparation. The RBR25 had the highest maximum torque during this preparation, which may suggest a risk for instrument deformation or fracture. Laboratory studies have shown that RB have greater resistance to cyclic fatigue when compared to that of WOG [1, 12–14]. However, the greater torque variation exhibited by the RBR25 in this study did not interfere with the instruments’ safety, as none deformed or fractured during their first use. This may justify the manufacturers’ indications that both instruments are single-use only.

A clinical study by Bueno et al. [15] found that the fracture indexes of the conventional WaveOne and Reciproc were low, and the instruments did not show any significant differences after reuse; however, the WOP fractured in one case, and the R25 had two cases. The highest torque for RBR25 found here cannot be associated with the RB fractures of the aforementioned clinical study, because they are two different instruments with distinctive characteristics.

Kim et al. [16] and Plotino et al. [17] demonstrated that Reciproc R25 instruments have a lower metallic mass and lower torsional resistance in contrast to WOP. In addition, Silva et al. [18] stated that although blue heat treatment increases the flexibility of this instrument, it lowers the torsional resistance and lowers the torque required for failure. This suggests that the maximum torque reached by the instrument may be related more to torsional resistance than to cyclic fatigue.

Reciproc Blue instruments have an “S” cross-section, while WOG files are parallelogram-shaped. Due to this instrument design, it is expected that WOG have a lower cutting power and, consequently, generate lower debris production [19]. Plotino et al. [20] and Özyürek et al. [21] stated that Reciproc files have a higher cutting efficiency than WaveOne files, possibly due to the differences in cross-sectional design and cutting angle, and the cross-sectional design is a more important indicator of reciprocating instrument cutting power. However, the results of this study indicated that, although the RB has greater cutting efficiency, its maximum torque was higher in the apical third when compared to that of the WOG, which can be explained by the difference in taper and cross-sectional design. The RBR25 file has a larger taper, which may remove more dentin during preparation, but requires greater torque to do so.

When comparing the canal thirds in the same instrument group, the WOGP and RBR25 had similar results. The maximum torque of both instruments was significantly higher in the apical third but was not significantly different when compared between the middle and cervical thirds. This result agrees with the reportedly higher number of instrument fractures that occur in the apical third [22] due to the anatomical configuration of the root canal, which has a smaller diameter and greater curvature in this region [23].

The root canal preparation time for the RBR25 was higher than that of the WOGP. This was possibly due to its greater conicity, which increases the difficulty of instrument progression into the root canal [24]. Because the WOGL and RBR40 files exhibited similar results in all analyses, it can be concluded that both were equal in safety and predictability during the lower molar distal canal instrumentations. These canals had lower maximum torque medians than the mesial canals, which suggests that molar preparation should begin in the mesial canals where increased instrument stress will occur. The WOGL and RBR40 have a higher calibration, and because of the lower torque generated, they are considered more resistant to preparation and can generate a possible sub-instrumentation of the distal canal using instruments with an initial diameter of 40 and 45 [25, 26].

Further studies should be performed to evaluate the degree of consistency of this method with more than one operator or even with unexperienced operator to test the reliability and reproducibility of the results, considering that measurements can be modified by the operator’s digital pressure and this might be related to the operator’s experience.

### Conclusion

Both instruments exhibited higher torque in the apical third and there were no significant differences between the instruments in the distal canal.

## Limitations

The limitations of this study can be attributed to the root canal geometry before instrumentation, considering that it is not possible to standardize the internal anatomy of the root canal. Furthermore, the use of an operator-driven instrument has the disadvantage of introducing operator bias.

## Abbreviations

WOG: WaveOne Gold
RB: Reciproc Blue
WOGP: WaveOne Gold Primary 25.07
RBR25: Reciproc Blue R25 25.08
WOGL: WaveOne Gold Large 45.05
RBR40: Reciproc Blue R40 40.06
RTTM: real-time torque monitoring
WL: working length
